# Intraosseous Venous Malformation of the Zygomatic Bone: Comparison between Virtual Surgical Planning and Standard Surgery with Review of the Literature

**DOI:** 10.3390/jcm10194565

**Published:** 2021-09-30

**Authors:** Raúl Antúnez-Conde, Carlos Navarro Cuéllar, José Ignacio Salmerón Escobar, Alberto Díez-Montiel, Ignacio Navarro Cuéllar, Giovanni Dell’Aversana Orabona, José Luis del Castillo Pardo de Vera, Carlos Navarro Vila, José Luis Cebrián Carretero

**Affiliations:** 1 Maxillofacial Surgery Department, Hospital General Universitario Gregorio Marañón, 28009 Madrid, Spain; comunicacionrac@outlook.es (R.A.-C.); jisalmeron@telefonica.net (J.I.S.E.); diezmontiel@gmail.com (A.D.-M.); nnavcu@hotmail.com (I.N.C.); hazamachado2@gmail.com (C.N.V.); 2 Maxillofacial Surgery Department, Università “Federico II”, 80131 Naples, Italy; giovanni.dellaversanaorabona@unina.it; 3 Maxilofacial Surgery Department, Hospital Universitario La Paz, 28046 Madrid, Spain; delcastillo6@hotmail.com (J.L.d.C.P.d.V.); rodrigator2001@hotmail.com (J.L.C.C.)

**Keywords:** vascular anomalies, zygomatic bone, virtual surgical planning, CAD-CAM technology, stereolithographic models, patient-specific implant, head and neck reconstruction, immunohistochemistry

## Abstract

Intraosseous venous malformations affecting the zygomatic bone are infrequent. Primary reconstruction is usually accomplished with calvarial grafts, although the use of virtual surgical planning, cutting guides and patient-specific implants (PSI) have had a major development in recent years. A retrospective study was designed and implemented in patients diagnosed with intraosseous venous malformation during 2006–2021, and a review of the scientific literature was also performed to clarify diagnostic terms. Eight patients were treated, differentiating two groups according to the technique: four patients were treated through standard surgery with resection and primary reconstruction of the defect with calvarial graft, and four patients underwent resection and primary reconstruction through virtual surgical planning (VSP), cutting guides, STL models developed with CAD-CAM technology and PSI (titanium or Polyether-ether-ketone). In the group treated with standard surgery, 75% of the patients developed sequelae or morbidity associated with this technique. The operation time ranged from 175 min to 210 min (average 188.7 min), the length of hospital ranged from 4 days to 6 days (average 4.75 days) and the postoperative CT scan showed a defect surface coverage of 79.75%. The aesthetic results were “excellent” in 25% of the patients, “good” in 50% and “poor” in 25%. In the VSP group, 25% presented sequelae associated with surgical treatment. The operation time ranged from 99 min to 143 min (average 121 min), the length of hospital stay ranged from 1 to 2 days (average of 1.75 days) and 75% of the patients reported “excellent” results. Postoperative CT scan showed 100% coverage of the defect surface in the VSP group. The multi-stage implementation of virtual surgical planning with cutting guides, STL models and patient-specific implants increases the reconstructive accuracy in the treatment of patients diagnosed with intraosseous venous malformation of the zygomatic bone, reducing sequelae, operation time and average hospital stay, providing a better cover of the defect, and improving the precision of the reconstruction and the aesthetic results compared to standard technique.

## 1. Introduction

Intraosseous vascular anomalies are uncommon [1], representing between 0.5–1% of intraosseous tumors [2]. More atypical are intraosseous vascular malformations involving the face, with 155 cases described in the scientific literature to date. The intraosseous involvement of the zygomatic bone is exceptional, with 64 cases published in the literature; of these, only 13 cases were venous malformations. It is common to find the indistinct use of both terms in the scientific literature, hemangioma and vascular malformation, as the terminology has not been standardized [3,4,5]. The treatment of these lesions comprises complete resection of the lesion and immediate reconstruction of the defect. The most common method used to reconstruct the defect is the use of an autologous bone graft, which provides good results. The increasing demand for aesthetic results as well as the improvement and refinement of procedures and techniques has enabled the application of virtual surgical planning to this field of facial surgery. Virtual surgical planning (VSP), the development of cutting guides using stereolithographic models (STL) and computer-assisted design and manufacturing (CAD-CAM) technology with a patient-specific implant (PSI) provide more predictable and accurate results [6].

The aim of the study was to compare the results achieved through standard surgery reconstructed with calvarial graft and VSP, CAD-CAM technology, STL models and a PSI. The specific aims were: (1) to compare the sequelae and complications with both techniques; (2) to evaluate the aesthetic results; (3) to compare the surgical time and mean hospital stay; (4) to compare the defect surface coverage through postoperative CT scan; (5) to review the literature in order to describe the usual presentation, study and treatment of these venous lesions and to clarify the diagnostic terms.

## 2. Materials and Methods

To address the research purpose, the authors designed and implemented a retrospective study with eight patients diagnosed with intraosseous venous malformation in the zygomatic bone between 2006 and 2021 at Hospital General Universitario Gregorio Marañon and Hospital Universitario La Paz, Madrid, Spain. Eight patients were diagnosed with intraosseous venous malformation and treated with complete resection and primary reconstruction. Four patients were reconstructed with calvarial bone graft (standard technique), and four patients were treated through VSP, cutting guides, STL models and PSI. The inclusion criteria were: (1) patients with a diagnosis of vascular malformation affecting the zygomatic bone; (2) patients reconstructed with standard technique with calvarial graft; (3) patients treated through VSP, STL models, cutting guides and PSI. The exclusion criteria were: (1) patients with prior surgery or surgical sequelae affecting the zygomatic region; (2) patients with vascular malformation with multiple involvement of several facial bones and/or extensive soft tissue involvement. The follow-up ranged from 2 years to 15 years. This study followed the Declaration of Helsinki on medical protocol and the study and review of the medical records, data collection and the subsequent analysis are endorsed by the Hospital Ethics Committee (protocol code maxilohgugm 04/2020).

All cases were studied by MRI and CT. In addition, in two cases, the study was completed by arteriography due to the high risk of bleeding. There was no tumor blush susceptible to embolization. In the group treated with standard surgery, four patients were reconstructed by means of a calvarial graft obtained in the same surgical procedure through a parietal approach after the zygomatic tumor had been resected. The malar bone defect was measured in the operative field and the calvarial graft was modeled to fit the defect. The graft was fixed with miniplates. During the first postoperative month, a CT scan was performed in all patients. Four patients were treated using VSP for the osteotomies design, cutting guides, STL models and titanium or polyether-ether ketone (PEEK) PSI. CT and MRI were performed for the preoperative study in all patients. In one patient, arteriography was performed without identifying any vessel suitable for embolization. A 3D reconstruction was obtained through preoperative CT scan and a remote connection was established with the engineering team (Maffinter Med.©, Madrid, Spain). The osteotomy cutting guides were designed using CAD-CAM technology manufactured with a computer numerical control (CNC) milling machine (Maffinter Med.©, Madrid, Spain). A surface and volume measurement of the bone defect secondary to resection was performed on the virtual model, and a custom-made titanium or PEEK prosthesis was designed for each patient. Special care was taken to ensure the correct fit, avoiding irregular edges or protrusions that could compromise the patient’s comfort or esthetic results. The optimal fit of the bone cutting guides and the PSI (Maffinter Med.©, Madrid, Spain) was checked on printed STL models. A subciliary approach was performed in two cases and subciliary and hemicoronal approach in two other patients. After exposure of the tumor, the osteotomy guides were adapted and tumor resection was performed with a piezoelectric scalpel. The PSI was adapted to the defect with excellent fit in all cases. It was fixed with titanium screws according to the manufacturer’s instructions. There was no evidence of irregular surfaces or inadequate adjustment in the treated cases. A control CT scan was performed during the first postoperative month.

The variables evaluated in this study were:

1. Defect surface coverage through postoperative CT scan: the Radiodiagnostic Department compared the defect covered with a patient-specific implant in the VSP group and the defect covered with a calvarial graft in the standard technique group to assess precision and accuracy of both reconstructions. Phillips IntelliSpace Portal V.11.1 software was used to calculate the defect after resection of the malformation and the percentage coverage obtained after reconstruction.
2. Operation time and length of hospital stay between both techniques.
3. Aesthetic result: aesthetic assessment was subjectively addressed by the patients regarding facial symmetry, facial scaring and facial projection and comparing the affected side with the non-affected side. The results were classified with scores 0 (poor), 1 (good) and 2 (excellent).
4. Sequelae and complications with both techniques.

### Review of the Literature

In order to establish the number of cases published in the literature with a diagnosis of zygomatic venous malformation of the zygomatic bone and to determine the most common surgical treatment, a review of the literature was performed. A complete search of reported cases, such as intraosseous vascular malformation and intraosseous hemangiomas of the facial skeleton, was performed in PubMed. Due to the indistinct use of terminology, the search was performed including the keywords “hemangiomas” and “vascular malformations”, which included venous malformations. A large number of cases have been reported interchangeably with one diagnosis or another due to the lack of immunohistochemical studies of the lesion endothelium. The search was carried out with the following algorithms:

- hemangiomas: (intraosseous [Title] OR mandibular [Title] OR osseous [Title] OR central [Title]) AND (hemangioma [Title] OR hemangiomas [Title]) AND (face [Title] OR midface [Title] OR facial [Title] OR zygomatic [Title] OR zygoma [Title] OR malar [Title] OR maxilla [Title] OR maxillary [Title] OR orbital [Title] OR orbit [Title] OR mandible [Title] OR jaw [Title] OR jaws [Title] OR bone [Title]).
- vascular malformations: (intraosseous [Title] OR mandibular [Title] OR osseous [Title] OR central [Title]) AND (malformation [Title] OR malformations [Title]) AND (arteriovenous [Title] OR venous [Title] OR vascular [Title]) AND (face [Title] OR midface [Title] OR facial [Title] OR zygomatic [Title] OR zygoma [Title] OR malar [Title] OR maxilla [Title] OR maxillary [Title] OR orbital [Title] OR orbit [Title] OR mandible [Title] OR jaw [Title] OR jaws [Title] OR bone [Title]).

The selected cases were filtered manually, eliminating articles related to single soft tissue involvement, publications of pathological or radiological interest without the contribution of clinical cases. A subgroup of venous malformations was also established (Appendix A). From each selected case, the following information was extracted: age, sex, location and size of the lesion, history of pain, ophthalmological alterations, history of trauma, radiological findings, surgical treatment, surgical approach, bleeding, type of reconstruction, performing immunohistochemical techniques, definitive diagnosis, follow-up and recurrence.

## 3. Results

### 3.1. Characteristics of the Sample

Eight patients underwent surgical treatment with a diagnosis of intraosseous vascular malformation located in the zygomatic bone. Four patients were treated surgically by standard technique in which reconstruction of the bone defect was performed with calvarial graft. Four patients received surgical treatment through VSP, CAD-CAM technology, STL models and PSI. In this group, two patients were reconstructed with titanium implants and two patients were reconstructed with PEEK implants indistinctly.

The mean age of the patients was 46.5 years. The average age of patients treated with standard surgery was 44.5 years and the average age for the group treated through virtual surgical planning was 48.5 years. Two patients reported pain as a symptom. All cases were studied with MRI and CT scans, although in two cases the study was completed with arteriography due to the possibility of a large diameter vessel that could be embolized prior to surgery. In all cases, CT scan showed a sclerotic tumor with a trabecular bone “honeycomb” pattern. The MRI findings consisted of a slightly expansive bone lesion hypointense in sequences enhanced in T1 and presented heterogeneous hyperintensity with hypointense areas in relation to thickened bone trabeculae in the T2 turbo spin-echo sequence. In the dynamic study T1, after the administration of contrast medium, a signal curve with progressive time-increase was identified in all cases. All this was suggestive of vascular malformation of low grade and venous nature in all the patients included in the study. In six patients, the resection was performed with a piezoelectric scalpel to minimize the risk of bleeding. In five patients, a combined hemicoronal and subciliary approach was used for tumor exposure, and in two cases, it was necessary to perform only a subciliary approach. One patient required a subciliary approach with a lateral canthotomy to provide complete resection of the malformation. At the end of the follow-up period, no local recurrence was observed in any patient.

### 3.2. Outcomes in Patients Treated by Means of Standard Surgery

**Figure 1:**
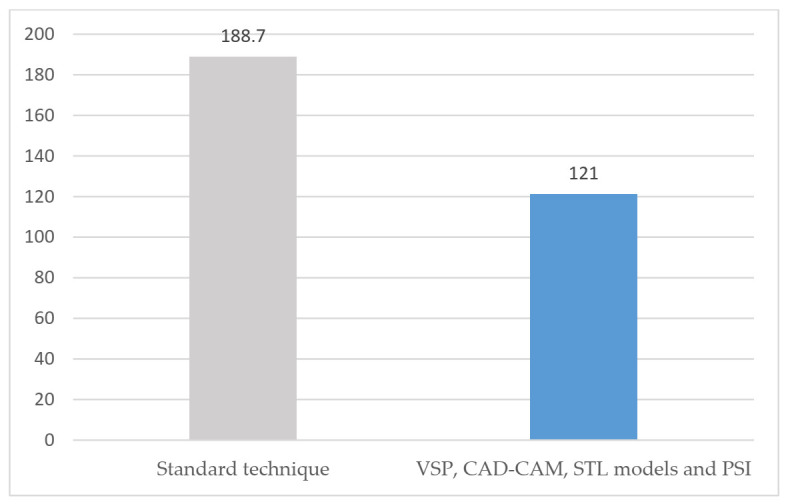
Surgical time (minutes).

**Figure 2:**
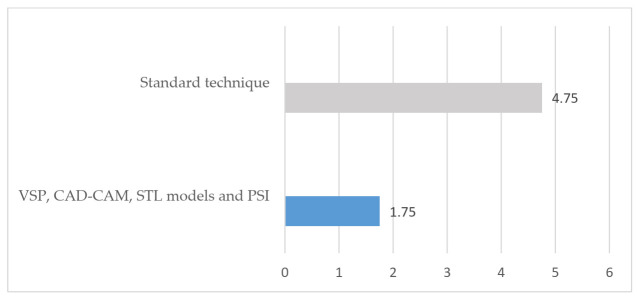
Hospital stay (days).

**Figure 3:**
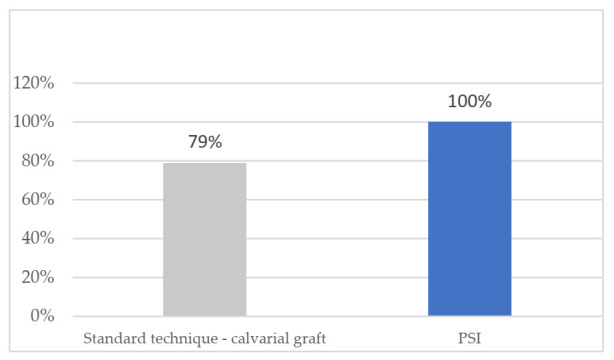
Coverage of defect (percentage).

In the group treated with standard surgery (Table 1), reconstruction was performed using a double calvarial graft harvested in the same surgical procedure through a hemicoronal approach when the zygomatic tumor had been resected. The malar bone defect was measured intraoperatively and the calvarial graft was modeled to reconstruct the defect. The average operation time for this group of patients was 188.7 min (range 175–210) (Figure 1). The average hospital stay was 4.75 days (range 4–6) (Figure 2). Postoperative CT scan showed a defect surface coverage of 79.75% (71–87) (Figure 3). No recurrences were observed during follow-up. The patients presented a scar on the scalp secondary to the harvesting of the calvarial graft as a sequela. In one patient (25%), lagophthalmos resulted as a sequela caused by the approach, which was corrected by canthopexy in a second surgical procedure. In one patient, a lateronasal incision was combined with a subciliary approach and lateral canthotomy. In another patient (25%), several months after surgery, the osteosynthesis material was partially removed due to discomfort reported by the patient. In all cases, the resected specimen was analyzed and an immunohistochemical study was performed with the result of intraosseous venous malformation. The aesthetic result was reported as excellent in one patient, good in two patients and poor in one patient.

### 3.3. Outcomes in Patients Treated by Means of VSP, CAD-CAM, STL Models and PSI

In the group of patients treated through VSP (Table 2), a surface and volume measurement of the bone defect secondary to the resection was performed on the virtual model, and a customized, patient-specific implant was designed for each patient. The optimal fit of the bone cutting guides and the PSI was checked on printed STL models. Subciliary approach was performed in two cases and the subciliary and hemicoronal approach in two patients. The average operation time was 121 min (range 99–143) (Figure 1). The average hospital stay was 1.75 days (range 1–2) (Figure 2). Postoperative CT scan showed 100% coverage of the defect surface (Figure 3). The esthetic results were reported as excellent in three patients and good in one patient. One patient developed a paresis of the frontal branch of the facial nerve due to possible accidental damage during the surgical approach. Histological and immunohistochemical analysis revealed an intraosseous venous malformation in all cases.

### 3.4. Case Presentation

**Figure 4:**
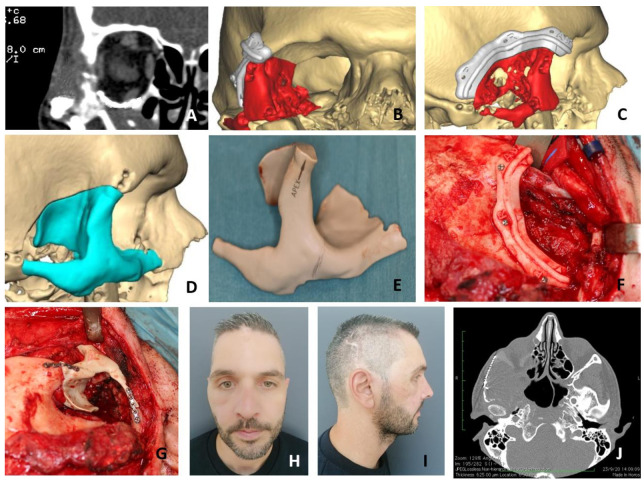
A 42-year-old male with a history of orbital floor fracture treated surgically with titanium mesh 7 years before being diagnosed during follow-up with a vascular malformation of the right zygomatic bone (**A**). Virtual surgical planning was performed for resection and reconstruction with a PEEK implant. Cutting guides were designed for resection (**B**,**C**). A customized PEEK PSI was specifically designed for the reconstruction (**D**,**E**). Due to the history of orbital floor surgery with a titanium mesh, it was decided to remove it and to extend the PSI to the orbital floor to avoid possible interferences between both reconstructions. A hemicoronal approach was performed and cutting guides were used to perform the resection (**F**). Once the malformation was removed, the PEEK PSI was placed with an excellent fit (**G**). The postoperative CT scan showed a correct placement of the PEEK PSI with an optimal aesthetic result (**H**–**J**).

**Figure 5:**
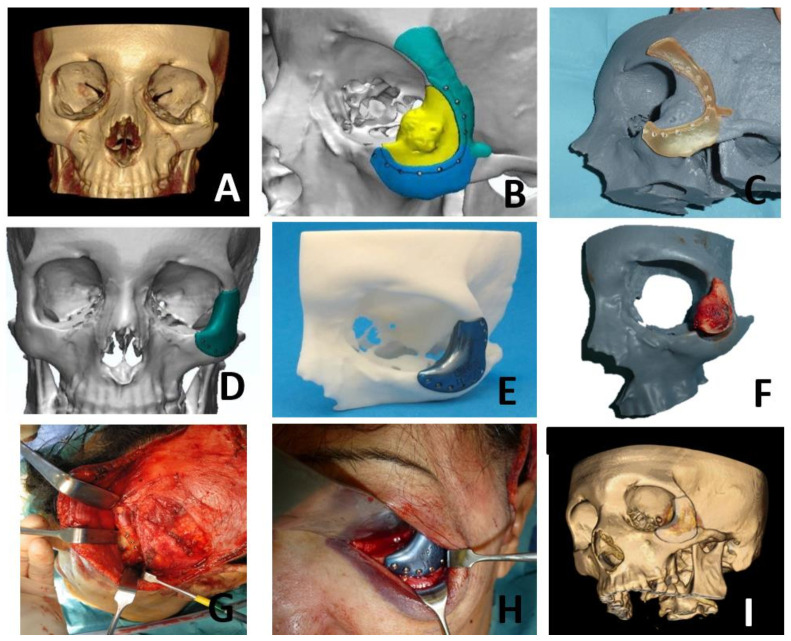
A 55-year-old woman, smoker, with a history of multiple sclerosis, reported a tumor of progressive growth and pain in the left zygomatic region. After study by CT, MRI and arteriography, the patient was diagnosed with intraosseous vascular malformation in the zygomatic bone (**A**). Virtual surgical planning was performed for resection and reconstruction with a titanium PSI. Cutting guides were designed for resection (**B**,**C**). A customized titanium PSI was specifically designed for the reconstruction (**D**,**E**). A hemicoronal and subciliary approach were performed and cutting guides were used to perform the resection (**F**–**H**). Once the lesion was removed, the titanium PSI was placed with an excellent fit (**H**). The postoperative CT scan showed a correct placement of the titanium PSI with an optimal aesthetic result (**I**).

**Figure 6:**
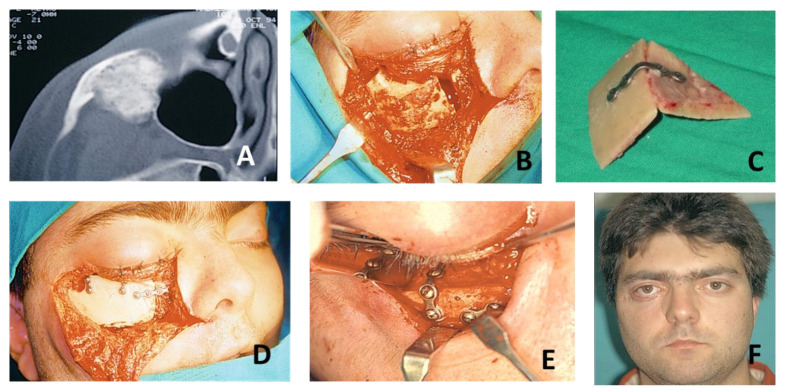
A 55-year-old male was referred from another hospital with a suspected intraosseous vascular malformation in the right zygomatic bone. After CT and MRI studies, the diagnosis was confirmed (**A**). Surgical treatment was performed using standard technique with a subciliary approach with external canthotomy and lateronasal incision (**B**) with complete resection of the tumor and primary reconstruction by means of a calotte graft obtained in the same surgical act (**C**). The calotte graft was adapted to the defect and fixed by means of miniplates and ostesynthesis screws (**D**,**E**). During follow-up, the patient reported discomfort in relation to the oteosynthesis material, so it was partially removed. Moreover, he presented lagosphthalmos as sequelae of the approach (**F**).

- Case Presentation 1 for Figure 4: VSP, Cutting Guides, STL Models, PEEK PSI.
- Case Presentation 2 for Figure 5: VSP, Cutting Guides, STL Models, Titanium PSI.
- Case Presentation 3 for Figure 6: Standard Surgery and Reconstruction with Calvarial Graft.

### 3.5. Literature Review and Clarification on Diagnostics Terms

With the screening of the data, it has been possible to establish that the most frequent anatomical presentation of intraosseous vascular malformations, within the facial skeleton, was the jaw, which accounts for two-thirds of all cases. The second site of involvement was the zygomatic bone, followed by maxillary, frontal, and other locations. Among the 64 cases affecting the zygomatic bone, only 13 were venous malformations (Appendix A); the distribution by sex showed a predominance of the female sex (females, 46; males, 18), representing almost 64% of the total. The prevalence was 3:1 in women. The age range of the patients included in the review was from 1 month to 73 years; the presentation was more frequent during the fourth decade of life. In the subgroup of venous malformations, the predominance was also female sex (females, 10; males, 13), with a range age from 15 to 66 years. There was a prior history of trauma in 14% of the patients (9 cases) and in 15% of patients diagnosed with venous malformation; it is necessary to clarify that in 35 of the remaining cases, a history of trauma was ruled out and, in another 21 cases, the patient was unaware of it or did not report it. The average time of development of the lesion was 25.7 months in general and 19.4 months for the subgroup of venous malformations. Almost 37% of the patients (22 cases) reported pain at the time of examination, although in some cases, it was described as “discomfort”. Nine patients (14%) previously presented ophthalmologic changes as a consequence of the tumor. Most patients, however, were asymptomatic. In the subgroup of venous malformations, there was a history of pain in 38% of the cases (five patients). There was a history of trauma in 15% of the cases (two patients). The most requested imaging test for the evolution of the lesions was CT, present in more than 70% of the cases. MRI was described in nine patients (14%), all of them after 2001.

The most frequent histologic diagnosis was hemangioma (79.6%, 51 cases). By subgroups, 31 hemangiomas, 15 cavernous hemangiomas, 4 capillary hemangiomas and 2 “mixed” cases were described. The diagnosis of venous malformation was reported in 13 cases (21.8%). Of the total cases in the literature review, only three cases (4.6%) specified histologic study and immunohistochemical techniques.

**Figure 7:**
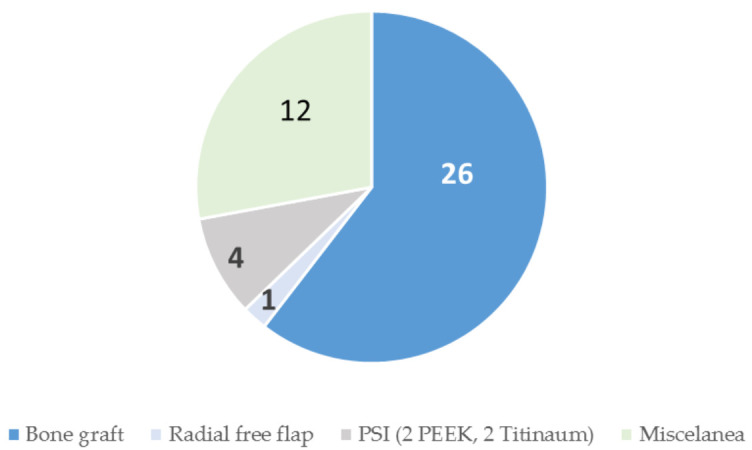
Surgical treatment reconstruction in vascular malformations of the zygomatic bone.

As for treatment, complete excision of the lesion was the gold standard (55 cases, 86%). An incisional biopsy was performed in eight cases, but in six of them, it was extended to complete excision. One patient refused surgical treatment. Among the patients operated by resection or excision, 78% (43 cases) underwent primary reconstruction; 26 cases received an autologous bone graft: calvarial graft (42%, 18 cases), iliac crest (11.6%, 5 cases) and rib (7.3%, 3 cases). In one patient (1.5%), reconstruction was performed with a radial osteofascial free flap. Only four cases (6%) received a specific implant customized for reconstruction. Two of them consisted of alloplastic implants: methylmethacrylate and polyetheretheretherketone (PEEK) implants, respectively (Figure 7). In the third patient, a specific titanium implant was fitted, but the authors have not reflected the surgical sequence or the type of planning. Another patient was reconstructed with a titanium PSI with good results and a description of the technique. The highest frequency of bleeding has been observed in cases who underwent biopsy (14%, nine cases) with respect to intraoperative bleeding (6.2%, four cases). No case of recurrence has been reported during the follow-up of the cases published in the literature.

**Figure 8:**
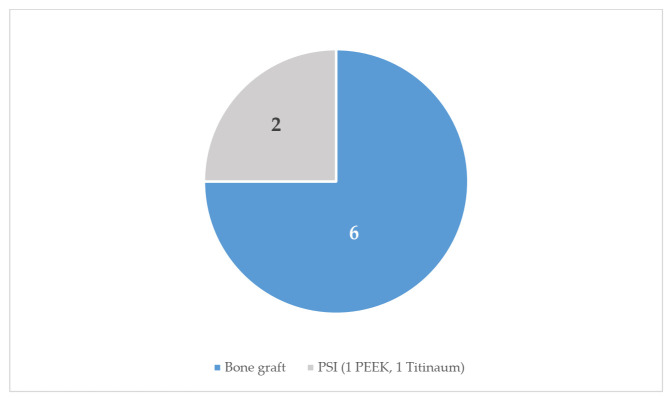
Surgical treatment reconstruction in venous malformations of the zygomatic bone.

The treatment used in the subgroup of venous malformation was mainly complete excision (eight patients, 61.5%). Partial resection was performed in two cases (15%), curettage of the lesion in one case (8.5%) and surveillance in two cases (15%). One case was reported with intraoperative bleeding. From 13 venous malformations, eight patients received reconstruction (Figure 8). Calvarial graft (38%, five cases) and iliac crest (7.6%, one case) were the two bone donor areas.

There was one case which received a customized alloplastic implant fixed with titanium miniplates and screws, while another patient received a titanium PSI though VSP, cutting guides developed with CAD-CAM technology and STL models.

## 4. Discussion

During the years 2006–2021, our group treated eight patients diagnosed with vascular malformation of the zygomatic bone. Primary reconstruction was performed in all patients. One of the objectives of this study was to compare the complications and/or sequelae associated with both techniques in patients diagnosed with vascular malformation of the zygomatic bone treated by standard technique and by VSP, CAD-CAM technology, cutting guides, STL models and PSI. In patients who underwent standard surgery, the following complications were obtained: dysesthesia of the donor area of the autologous calvarial graft (three cases, 75%), pain in the area of the intervention that required intravenous analgesic treatment during hospital stay (one case, 25%) and one case of lagophthalmos (25%). Since in this group of patients the resection was performed without cutting guides, the exposure of the surgical sites was wider to visualize the limits of the resection as well as to allow the use of different surgical tools in a small space. In one patient, lagophthalmos occurred due to the need to widen the subciliary approach by lateral canthotomy for better tumor exposure. In this patient, it was also necessary to partially remove the ostosynthesis material due to discomfort related to self-palpation. In the case in which a hemicoronal approach was required, the harvesting of the calvarial graft did not involve added morbidity. However, in the other three cases, the patients reported dysesthesia related to the donor site scar. It is necessary to clarify that, in one of the patients, there were no sequelae due to surgery, and in none of the cases recurrence of the vascular lesion was observed.

In relation to the patients treated through virtual surgery, frontal branch paresia was observed in one patient (25%) due to the hemicoronal approach, although it was transitory and complete recovery was observed 6 months after surgery. In the rest of the cases treated using VSP, CAD-CAM technology, STL models and PSI, there were no sequelae. We consider that the decrease in morbidity in this group of patients is due to: (1) a reduction in the size of the surgical wound with the advantage of better control of tumor resection by using cutting guides [7]; (2) the avoidance of an approach related to the calvarial donor site; (3) the better anatomical fit of the customized implant [8,9]. Total coverage of the defect was observed in the patients treated through VSP, cutting guides and PSI, which was also more anatomical and did not present angles or peaks that could compromise the integrity of the reconstruction [6].

Although the most frequently reported complication in the literature was intraoperative bleeding, only one case [4] out of 64 presented with a venous malformation. In our case series, it was not evident in any of the patients. This is probably due to the low flow of this type of lesion. No other complications or sequelae have been reported in the literature, which may be due to the absence of follow-up or to studies that only describe the findings of the clinical case.

The aesthetic results were good with both techniques; however, when evaluating the results observed in both groups, we found significant differences. In total, 75% of the patients who underwent virtual surgery reported an excellent esthetic result and 25% referred a good aesthetic result. These results were also assessed by the surgical team, who evaluated the result according to the presence or absence of sequelae, visible scars and facial symmetry. One patient, affected postoperatively by frontal branch paresia, assessed the esthetic result as good. After recovery of the paresia of the frontal branch of the facial nerve, the result was excellent.

In the group treated with surgery according to the standard technique, 50% of the patients (two cases) reported a good result after surgery, one patient (25%) reported a poor result and one patient (25%) reported an excellent result. The patients who reported a good result presented adequate facial aesthetics; however, they reported discomfort in the graft donor area. The patient with a poor aesthetic result had presented lagophthalmos as a sequelae of an unguided tumor resection approach and, in addition, had discomfort after reconstruction due to palpation of part of the osteosynthesis material under the skin. Additionally, in this case, we found an advantage in the use of the PSI, which, in addition to providing a more anatomical reconstruction, does not present irregular surfaces, edges or protrusions of the osteosynthesis material that could compromise the reconstruction or the patient’s comfort. [6,10].

The use of titanium PSI or PEEK PSI was indistinct. In general, polyether-ether-ether-ketone prostheses have high resistance and are biocompatible; they are radiolucent, and thus, they produce minimal artifacts on MRI. It is a lightweight material that can undergo changes during surgery with standard instruments. Titanium PSI is characterized by a higher strength and a modulus of elasticity 30 times higher than PEEK PSI. It is a radiopaque material with excellent biocompatibility and osseointegration characteristics. Due to its hardness, it is difficult to make intraoperative changes to the product supplied by the commercial manufacturer [11].

The third objective of this study was the comparison between the operation time required to perform each of the techniques and the mean hospital stay among the patients. Patients treated with standard techniques had a mean operation time of 188.7 min (range 175–210) and patients treated with VSP, cutting guides developed with CAD-CAM technology, STL and PSI models, had a mean operation time of 121 min (range 99–143). The 30% reduction in operative time was a result of faster and more precise resection using CAD-CAM cutting guides and testing on STL models after VSP. This reduction in operation time also meant a reduction in anesthesia time and potential risks [9].

The average hospital stay of patients who underwent standard surgery was 4.75 days (range 4–6); however, the average hospital stay of patients who underwent virtual surgery was 1.75 days (range 1–2). This reduction in the number of days of hospitalization was the result of the decrease in surgical morbidity, avoiding the problems derived from harvesting the calvarial bone graft. In addition, since the approach was more limited due to the greater safety of the area to be resected through the use of cutting guides, postoperative pain was reduced in all cases [6,9]. The reduction in surgical time and hospital stay resulted in considerable savings in hospital costs; however, we should not forget the costs associated with VSP, the development and manufacture of the models and cutting guides and the design and production of the customized implants [9]. Further studies should be conducted to evaluate the costs of comparing the two techniques in a specific healthcare system. These data could not be compared with other studies, since there are no specific publications on cases of zygomatic venous malformation in which surgical time has been evaluated.

The fourth objective of the study was to evaluate and compare the reconstruction of the defect after resection with each technique. For this purpose, the Radiodiagnostic Department performed a volumetric measurement of the defect developed in the zygomatic bone in the postoperative CT scan among the patients treated. The CT images were subtracted by component to measure the percentage of the defect covered by reconstruction, either PSI or calvarial graft. In all patients to be treated with VSP, CAD-CAM cutting guides and titanium or PEEK PSI, 100% defect coverage was observed. In patients treated with the standard technique, the defect created after resection was covered by 79.75% (range 71–87). The total defect coverage obtained in patients treated with VSP was a result of (1) a more precise resection through the use of cutting guides; (2) a defect with more uniform edges previously planned; (3) the verification of the good PSI fit in STL models before surgery. This correct PSI fit is one of the main advantages of treating this type of lesion using VSP, CAD-CAM-developed cutting guides, STL and PSI models, as more restricted resection defects are created, resulting in smaller surgical approaches with faster healing [6,7,8].

The last objective of our study was to review similar cases published to date and to clarify the terms used in the scientific literature for the diagnosis of vascular malformations, especially those affecting the zygomatic bone. We also reviewed the clinical presentation, study and treatment of these lesions. Thus, 155 total publications were obtained, which included intraosseous hemangiomas and intraosseous vascular malformations related to facial bones, of which 64 were involved the zygomatic bone. Among the 64 published cases, there are 24 that were exposed as vascular malformations (not specified), 27 as hemangiomas and 13 as venous malformation, although most of the earlier ones were reported an intraosseous hemangioma.

The current vascular anomalies classification was adopted by the ISSVA in 1996, incorporating the previous classification of Hamburg (1988) and based on the classification proposed by Mulliken and Glowacki in 1982 [3]. As noted, hemangiomas are proliferative lesions with a benign tumor character with a pattern of progressive growth and subsequent involution [4]. These may be childhood or congenital hemangiomas [12,13,14,15,16,17]. These lesions have a typical characteristic: their appearance is fully developed at the time of delivery, which implies that their proliferative phase has been carried out exclusively “in utero” [18,19,20].

On the contrary, vascular malformations are lesions resulting from the remainder of vascular embryological tissue, which has progressive growth without involution and may be affected by hormonal factors, such as puberty or pregnancy, undergoing accelerated growth under these circumstances [4,21,22]. Within them, venous malformations represent the most frequent peripheral vascular malformation [23]. Their origin is a congenital venous ectasia of a physiological venous territory or the abnormal development of the efferent vascular tree [24,25]. They are usually located in the head and neck (40%), extremities (40%) and trunk (20%) [21]. Many venous malformations have been incorrectly referred to as “hemangiomas”, although the growth pattern, therapeutic attitude and prognosis of both lesions are completely different [5]. Thus, they are present at birth, although they are not evident until weeks, months or years later, they do not disappear spontaneously unlike hemangiomas and they grow in parallel with the physiological development of the child.

Most of the articles about vascular malformation affecting the zygomatic bone did not present an immunohistological justification for the diagnosis provided, so it is impossible to specify whether they were really intraosseous hemangiomas, venous malformations or other vascular malformations. Moreover, before 1982, it is common to find allusions to “arteriovenous malformation” as a synonym for hemangioma [4]. Even so, there are much more recent publications with this terminology that lead to error and several articles have even been obtained in which the pathological outcome differs from the title of the manuscript. It is noteworthy that despite having the binary classification since 1982, it took 7 years to find the first case published [4,17]. Before 1996, more than 90% of cases were presented as intraosseous hemangioma. Since 1996, year of publication of ISSVA classification, the use of erratic and confusing terms affects almost half of the published cases. Although the review of the literature includes cases published since 1950, there was no diagnosis of “venous malformation” until 1991, which suggests different study techniques for the sample.

From the 64 cases of intraosseous vascular lesions of the malar bone reported in the literature, only 13 corresponded to intraosseous venous malformation of the zygomatic bone. A large number of patients have debuted with zygomatic asymmetry or bone tumor but, generally, they were asymptomatic. In the group of patients that presented symptoms, the most frequent was pain, followed by ophthalmological alterations [26,27]. There was a history of trauma in 15% of patients with venous malformations, despite having been postulated as one of the most common causes of intraosseous hemangiomas [28,29], which also leads us to believe that the erroneous terminology used in many publications may be responsible for this lack of relationship. Of the remaining cases, nine of them ruled out a history of trauma and two patients did not know. This form of clinical presentation is consistent with the patients treated by our group. However, none of the eight patients in our study had a history of trauma and two patients reported pain at the time of diagnosis. Only three of the thirteen cases published as venous malformation of the zygomatic bone were subjected to immunohistochemical study. The eight cases presented in this work were studied in this way, so the diagnosis of intraosseous venous malformation is certain.

The imaging test of choice for the study of intraosseous venous malformations is CT, being the most requested by physicians who assumed the diagnosis and treatment of reported cases (84%). This is the test is of choice because of its ability to distinguish the bone cortex from the trabecular portion more accurately than other techniques; however, it is not the most accurate when defining soft tissue involvement [2]. The most frequent descriptions of the radiographic appearance of the lesions were “honeycomb”, “soap bubbles” or “sun rays” [27,30]. MRI provides data mainly on soft tissues, but is limited to assess intraosseous lesions. In general, hyperintensity of the lesion in T1 and hypointensity in T2 can be seen, although these findings are also consistent with intraosseous lesions, such as odontogenic cysts, ameloblastoma, etc. Some authors defend its use instead of the CT, since the enhancement of the lesions in T2 provides key data in the diagnosis [31]. CT scans and MRI together with the three-dimensional reconstructions allow a more precise diagnosis of the lesion, its size and anatomical relationships. Arteriography can be used when the possibility of advantageous endovascular therapy is anticipated prior to surgery or as a definitive treatment, mainly in high-flow lesions. In the cases reviewed, selective embolization was rarely carried out, as well as in the cases of venous malformation provided, so its use seems limited [2].

Given the suspicion of vascular lesion, the biopsy is contraindicated due to the risk of bleeding, although if the involvement is soft tissue is substantial, some authors defend the possibility of performing a fine needle aspiration (FNA) [1]. In the case of the patients, the possibility of biopsy was not considered.

Contemplating the range of therapeutic options for these types of injuries in the literature, multidisciplinary management is considered the gold standard [4,32]. Surveillance may be an option in asymptomatic cases [33]. However, only two cases of venous malformation were handled in this way, and years later, they received surgical rescue due to the progressive growth of the lesion. Surgical treatment is the most adopted option by the authors, mainly through complete resection [29,33,34]. While management may differ in vascular anomalies that exclusively affect soft tissues, in intraosseous malformations, it seems to be the most appropriate option because it has shown the lowest recurrence rates. In some cases, partial resections and curettage have been performed. In cases of recurrence, complete resection was done. No recurrences have been reported of the cases that were treated by complete resection [35]. In the cases treated by our group, complete resection of the lesion was performed using piezoelectric scalpel to minimize bleeding due to the cavitation effect and to reduce the risk of associated lesion of the orbital content. The theoretical risk of soft tissue injury when performing complete resection is greater, although there have been no complications in the cases studied [36].

Regarding the ways of reconstruction, there is no consensus for the indication or the type; however, as these are resections with an important aesthetic sequel, most authors have chosen to do so. The lack of reconstruction can lead to a contraction of soft tissue that is difficult to correct with deforming sequelae [35]. Primary reconstruction is the most frequent option. Reconstruction with autologous bone is commonly used. Calvarial graft (38%, five cases) [27,29,35,36,37,38,39,40,41,42,43,44,45] and iliac crest (7.6%, one case) [1,46,47,48,49] are the two most frequent bone donor areas used; in the treatment of vascular malformations with the same location, rib (three cases) is the third most used autologous graft [50,51,52].

There is one case which received a customized alloplastic implant fixed with titanium miniplates and screws [53]. The use of custom alloplastic implants has the advantage of reducing the surgical time and eliminating morbidity in the donor area [44,53]. One patient received a titanium PSI though VSP, cutting guides developed with CAD-CAM technology and STL models [54].

The management and reconstruction methods seem to be less in venous malformations with respect to vascular malformations of the zygomatic bone in general (Figure 7 and Figure 8). This is due to the smaller number of published cases of venous malformations, since the management of both lesions is the same.

This work aims to compile all the cases published to date in the scientific literature with a diagnosis of intraosseous vascular malformation of the zygomatic bone in order to establish the diagnostic and treatment method. The investigators compare the differences between standard surgery and surgery through VSP, STL models, cutting guides and PSI. To date, although the main limitation of this study is the sample size given the low prevalence of this type of tumor in this location, this is the first comparative study of both techniques in the scientific literature. In addition, all samples obtained from patients treated by our departments have been subjected to immunohistochemistry techniques for the correct diagnosis. Therefore, further studies are required in the future to corroborate the advantages of guided surgery in a larger population.

## 5. Conclusions

The treatment of choice for intraosseous venous malformations involving the zygomatic bone is complete resection and primary reconstruction. It does not differ from the vascular malformations with the same location. The multistage implementation of VSP with the use of STL models, CAD/CAM cutting guides and PSI—titanium or PEEK—for zygomatic defects offers reconstructive accuracy, which previously depended on surgeon experience and intraoperative trial and error using 2D imaging modalities and autologous bone grafts. It increases resection accuracy, provides better reconstruction volume and improves operative efficiency with reduced complication rates and hospital stay. Moreover, it offers excellent aesthetic outcomes. The use of appropriate terminology, communication of clinical data between the surgeon and the pathologist and the use of immunohistochemical techniques in the processing of the specimen are necessary for the correct diagnosis and management of intraosseous vascular malformations.

## Figures and Tables

**Table 1:** Patients treated by means of standard surgery—calvarial graft.

	Age /Sex	Size cm.	Evolution	Clinics	Aesthetic Result	Histology	Sequelae/ Recurrence	Surgical Time (min.)	Coverage of Defect (%)	Hospital Days	Follow-Up
Case 1	35/M	1.5	10y	Pain	Good	Vascular malf.	Scalp scar dysesthesia, Pain/N	210	71	4	15y
Case 2	41/F	2	20y	No	Good	Venous malf.	Scalp scar dysesthesia/N	180	79	6	11y
Case 3	55/M	1	10m	No	Poor	Venous malf.	Scalp scar dysesthesia. Lagophtalmos/N Discomfort due to osteosynthesis material	175	87	5	10y
Case 4	47/F	1.5	7y	No	Excelent	Venous malf.	N/N	190	82	4	6y

**Table 2:** Patients who underwent VSP, CAD-CAM, STL models and PSI.

	Age /Sex	Size cm.	Evolu-tion	Clinics	Aesthetic Result	Histology	Sequelae/ Recurrence	Surgical Time Min.	Coverage of Defect %	Hospital Days	Follow-Up
Case 1	55/F	1.5	20y	Pain	Excelent	Venous malf.	N/N	143	100%	2	5y
Case 2	60/F	2	20y	No	Good	Venous malf.	Frontal paresia /N	124	100%	2	3y
Case 3	39/M	1	2y	No	Excelent	Venous malf.	N/N	99	100%	1	3y
Case 4	40/F	1.2	10y	No	Excelent	Venous malf.	N/N	118	100%	2	2y

## Data Availability

The data presented in this study are available on request from the corresponding author. The data are not publicly available due to data protections regulations.

## Abbreviations

CAD/CAM	Computer-Aided-Design/Computer-Aided-Manufacturing
CNC m.m.	Computer Numerical Control milling machine
CT scan	Computed tomography scan
ISSVA	International Society for the Study of Vascular Anomalies
MR	Magnetic Resonance
PEEK	Polyether-Ether-Ketone
PSI	Patient Specific Implant
STL model	stereolithographic model

## Appendix A

**Table A1:** Venous malformations of the zigomatic bone. M: male; F: female; R: right; L: left; Y: yes; N: no; y: year; m: month

	Author (Year of Publication)	Age/Sex	Side, Size	Duration	Pain	Ocular Findings	Traumatism	Imaging	Management	Surgical Approach	Reconstruction	Bleeding	Follow-Up, Recurrence	Inmunohistochemistry
1	Yueh-Bih Tang Chen et al. (1991) [38]	44/F	R/3 × 2 cm	1 y	Y	N	N	X-rays: radiolucent lesion	Excision	Coronal	Cranial bone graft	N	6 y, no recurrence	?
2	Srinivasan et al. (2009) [8]	66/F	R, 3 × 3 cm	4 y	N	N	N	CT: radiating spoke wheel	Excision	?	Not required	N	2.5 y, no recurrence	GLUT-1 negative
3	Defazio et al. (2012) [29]	58/F	R, 1 × 1.5 cm	2 y	N	N	Y	CT: sunburst; MRI: T2 with high signal intensity	Incisional biopsy; Surveillance	?	Not required	?	?	GLUT-1 negative
4	Defazio et al. (2012) [29]	53/F	R, 1 × 1.8 cm	?	Y	?	N	CT and MRI consistent with dx of intraosseous venous malformation	Initial incisional bi- opsy non-diagnos- tic; Surveillance; Complete resec- tion 2 y later	?	Split calvarial bone graft	?	?	?
5	Defazio et al. (2012) [29]	49/M	R, 1 cm	6 mo	N	N	N	CT: honeycomb	Complete resection	?	Split calvarial bone graft	?	?	?
6	Colletti et al. (2014) [27]	?/F	?	?	?	?	?	CT:sunburst pattern	En bloc resection	Lower lip incision	Split calvarial bone graft	?	?	?
7	Colletti et al. (2014) [27]	?/F	?	?	?	?	?	?	En bloc resection	Lower lip incision	Split calvarial bone graft	?	?	?
8	Xiuling Huang (2017) [1]	35/F	L/2.5 × 2.5 cm	7 y	N	N	N	CT: sunburst pattern of radiating trabeculae with intact cortices	Excision	Lower-eyelid incision	free iliac bone graft and minititanium plate	N	3 y, no recurrence	?
9	Xiuling Huang (2017) [1]	41/F	L/1.5 cm	2 y	N	N	N	CT: sunburst pattern of radiating trabeculae with intact cortices	Partial resection	Lower-eyelid incision	N	N	2 y, no recurrence	?
10	Xiuling Huang (2017) [1]	44/F	L/2 cm	3 m	Y	N	N	CT: well defined bone eminence	Partially resected	lower eyelid incision	N	N	12 y, no recurrence	?
11	Xiuling Huang (2017) [1]	49/F	R/1 cm	4 m	N	N	N	CT: well-defined radiolucency with trabecular density inside	Aggressive curettage	?	N	N	7 y, no recurrence	?
12	Zoltán Fábián et al. (2018) [4]	15/M	L/100 cm^3^	4 y	Y	Y, left eye dislocated to medial and cranial, mildly limited mobility	Y	CT: expanded zygomatic bone	*Preoperative embo- lization*; Complete resection	Weber-Fergusson -Dieffenbach incision	Patient Specific Implant	Y, during biopsy Y, moderate	?	?
13	Antúnez-Conde et al(2021) [54]	55/M	L/1.5 cm	Many years	Y	N	N	CT: "honeycombed osseous lesion", MRI	Excision	Coronal and subciliary	Patient Specific Implant	N	6 m, no recurrence	GLUT-1 negative
